# The Paradox of Tau and RNA-Binding Proteins: How Adaptive Stress Granule Regulation Becomes Pathological with Aging

**DOI:** 10.3390/cells15171596

**Published:** 2026-09-02

**Authors:** Benjamin Wolozin, Merci Best, Madhav Ellini, Yuran Ma, Sojung Bok, Dylan Hwang, Carolina Filipponi

**Affiliations:** Department of Anatomy and Neurobiology, Avedisian School of Medicine, Boston University Chobanian, Boston, MA 02118, USA

**Keywords:** TDP-43, MAPT, biological condensate, stress granule, membraneless organelle, liquid–liquid phase separation, oligomeric tau, translational stress response, protein aggregation, RNA metabolism

## Abstract

**Highlights:**

**What are the main findings?**

- Stress induces phase-separated biomolecular condensates (stress granules) that serve as nucleation sites for the aggregation of neurodegenerative disease-linked RNA-binding proteins.
- Stress-mediated phosphorylation stimulates tau oligomerization, which normally functions to stabilize stress granules. This process becomes pathologically amplified in disease, leading to excessive stress granule accumulation.

**What are the implications of the main findings?**

- Reducing biomolecular condensates in stress and disease could provide a general effective therapeutic approach for multiple neurodegenerative diseases.
- Translational stress pathways, such as eIF2 and eIF3, are specific pathways being pursued for reducing levels of stress granules, aggregated RNA binding proteins and oligomeric Tau.

**Abstract:**

RNA-binding proteins (RBPs) are a large class of proteins that form biological condensates to facilitate their functions. Chronic stress, such as occurs in neurodegenerative diseases, stimulates persistent accumulation of particular RBP condensates as part of the translational stress response, termed stress granules (SGs). These persistent SGs serve as a nidus for aggregation of RBPs to form pathologies that appear in neurodegenerative diseases, such as the occurrence of Tar DNA Binding Protein (TDP-43) in Amyotrophic Lateral Sclerosis. Many of the RBPs that accumulate in SGs are also associated with mutations that are linked to neurodegenerative diseases. The microtubule-associated protein tau is the major intracellular pathology that occurs in Alzheimer’s disease. Tau is phosphorylated with stress, whereupon it functions to regulate SG biology; conversely, SGs serve as a crucible for the accumulation of toxic oligomeric tau. The regulation of stress by tau is an inherent part of biology that normally occurs during development and hibernation; however, with aging it becomes pathological, possibly because of the reduced proteostasis associated with aging.

## 1. Introduction

Individuals in countries with technologically advanced healthcare often live well past 65 years, which is the classic demarcation of old age. Living into the geriatric years, however, sets the stage for increased risk of neurodegenerative diseases, such as Alzheimer’s disease (AD), Frontotemporal Dementia (FTD), Vascular Dementia, Parkinson disease (PD), Amyotrophic Lateral Sclerosis (ALS), Spinocerebellar Ataxias (SCAs) and other diseases [1,2]. Mutations in a wide variety of genes are associated with these diseases, but they fall into broad categories of proteostasis, inflammation and RNA metabolism. These diseases appear to share many similar processes as the body adapts and responds to the disease. Notably, most of these diseases are defined by unique hallmark proteins that accumulate as protein aggregates as well as mutations directly linked to these proteins, indicating that dysfunction of the protein is sufficient to cause disease [1,2]. Many of these hallmark protein aggregates participate in RNA metabolism, which includes the biology of biomolecular condensates. We hypothesize that the persistence of biomolecular condensates during stress can lead to protein aggregation. One of the goals of this review is to present how tau pathophysiology arises from normal biological processes occurring as part of RNA metabolism; stress causes tau hyperphosphorylation and oligomerization, which act to regulate stress granules (a type of biomolecular condensate) and the translational stress response. Protein aggregates produced by RNA-binding proteins (RBPs) or tau appear to disrupt many functions, leading to a state of chronic stress that broadly affects cell biology. The aggregates interfere with many processes, causing dysfunction of the autolysosomal pathway, mitochondrial pathway, lipid dishomeostasis and inflammation [3,4,5,6,7,8,9]. In parallel, the body adapts protein synthesis to match the reduced energy and nutrient production. All of these changes occur upon a baseline of increasing senescence. These processes impact the cell to produce a chronic stress response. Thus, a comprehensive understanding of disease needs to consider how the biology of chronic stress contributes to protein aggregation and dysfunction of RNA metabolism.

## 2. The Evolution of Pathology in Neurodegenerative Disease

There is a strong consensus among disease researchers that the accumulation of protein aggregates drives neurodegeneration, which is why so much of the field focuses on proteostasis [2]. Every neurodegenerative disease is associated with one or more hallmark protein aggregates, which represents a characteristic pathological feature of the disease. Genetic studies have identified mutations that enhance aggregation for the vast majority of these hallmark pathological proteins [2]. For example, mutations in APP cause AD and lead to Aβ aggregates that are a hallmark of AD [10]. Mutations in MAPT cause FTD–Tau and lead to the accumulation of Tau aggregates (as neurofibrillary tangles) [11]. Mutations in α-Synuclein cause PD and lead to the accumulation of α-Synuclein as Lewy bodies [12,13,14]. Mutations in TDP-43 are linked to ALS and TDP-43 aggregates [14,15]. Mutations in Huntingtin cause Huntington’s disease and HTT aggregates [16]. This is true for many other mutations.

Our understanding of the nature of a misfolded or aggregated protein has advanced in step with maturation of the field of neurodegenerative diseases. The aggregate proteoform occurring in the hallmark aggregates is classically thought to be the key species for understanding the process of neurodegeneration. This hypothesis has led to important work characterizing the structures of these protein aggregates by Cryo-EM. These approaches have yielded detailed images of the structures of these aggregates [15,16,17,18,19,20,21]. Cryo-EM reveals the mechanisms of stacking that yield the fibrils. The Cryo-EM images also reveal variation in the precise molecular bonds for a particular aggregating protein that differ by disease type [15]. Thus, although the same region of MAPT aggregates form β-sheets in all tauopathies, the structures of the MAPT differ among the different types of tauopathies, including AD, FTD–Tau, Corticobasal Syndrome and Chronic Traumatic Encephalopathy [15,17,22,23,24].

This clearly defined demarcation of disease-related aggregates could be an oversimplification that masks the actual heterogeneity of aggregates. The focus on pathological aggregates also distracts attention from smaller oligomers that do not form the large solid structures observed by neuropathologists but in many cases represent the most toxic elements in disease. One clear example of amyloid fiber heterogeneity appears in Cryo-EM studies of TDP-43, which form heteromeric filaments with Annexin 11 (ANXA11). TMEM106b aggregates can occur as mixed aggregates containing TMEM106b, Annexin-7 and/or TAF15 [20]. More generally, isolation of insoluble fractions from AD brain reveals an abundance of RBPs, as well as chaperones, which indicates a widespread process or protein aggregation occurring in neurodegenerative diseases, although most of these aggregates are amorphous in structure and do not form structurally defined amyloid fibrils [25,26]. The biological processes leading to this aggregation could arise in part from the formation of RNA granules through the process of liquid–liquid phase separation (LLPS), which is discussed later in this review.

Ascribing disease mechanisms to rigid amyloid aggregates arises from the connection of neurodegenerative disease genetics with the presence of protein aggregates that have β-sheet fibrils, such as β-amyloid and tau. These β-sheets are detected by amyloid sensing dyes, such as Thioflavin-S/T and Congo-Red, and thus have been central to the language of neuropathology for the past century. However, it is also clear now that many neurodegenerative diseases arise from aggregating proteins that do not form β-sheet structures and were hidden from the view of neuropathologists until antibodies were made to identify these proteins (in some cases aided by discovery-based mass spectrometry) [18,27].

The mechanisms leading to protein misfolding and aggregation appear to cover proteins that form intracellular amorphous aggregates and proteins that form intracellular, structurally defined amyloid aggregates (e.g., containing β-sheet structures, such as tau). Fibrillar aggregates containing tau represent the end stage of aggregate evolution. During stress, tau misfolds and then forms oligomers that can have many different structures; disease-related phosphorylation of tau accelerates this process, which might arise by biological design. Multiple studies have shown that these tau oligomers are more toxic to neurons than tau fibrils [28,29,30,31,32]. In addition, the speed of disease progression in tauopathies correlates better with the load of tau oligomers than the load of fibrils [31,32]. Thus, mechanisms of neurodegenerative disease must account for the wide array of oligomers and protein aggregates that accumulate in the brain.

The aggregates cause damage by three general mechanisms. First, some aggregates cause toxicity by sequestering essential proteins. TDP-43 provides a good example of this. An important function of nuclear TDP-43 is to suppress inappropriate splicing, termed cryptic splicing [33]. Cytoplasmic translocation of TDP-43 and sequestration of TDP-43 in cytoplasmic aggregates reduces the availability of free TDP-43, which results in deleterious cryptic splicing [33,34,35,36,37].

Misfolding, translocation and/or aggregation of proteins like TDP-43 are modulated by heat shock proteins (HSPs) and chaperones. The HSP70 family contains 13 genes. HSPA8 is expressed constitutively, while most others localize to particular organelles or tissues and are induced during stress [38,39]. A large group of chaperones modulate HSP70 function and mediate the role of ATP in preventing protein misfolding. HSP90 proteins are a different class of proteins that reduce protein misfolding and aggregation. The HSP90 family of proteins constitutes 1% of cellular proteins and interacts with client protein for directed proteostasis [38,39].

Progression of neurodegenerative diseases causes persistent proteostatic stress. Some HSPs are pulled into the insoluble fraction because of their binding to aggregated proteins. Other HSPs become dysfunctional because the accumulation of protein aggregation changes the availability of chaperones for their networks, producing large, poorly functioning chaperone scaffolds that fall into the general category of the epichaperome [38,39].

Many protein aggregates cause damage, through a gain of function mechanisms in which small, more soluble oligomers interfere with the normal functions of existing protein. These mechanisms of toxicity caused by protein aggregates are highly pleiotropic, and include disruption of RNA metabolism, disruption of mitochondrial function and disruption of synaptic function. Third, fibrillar protein aggregates can interfere with the functions of membranous organelles by puncturing the membranes; this puncturing mechanism is well documented for both the nuclear and lysosomal membranes [30,40,41].

The broader genetics of neurodegenerative diseases reflect the biological processes that contribute to the proteostasis factors that can regulate the accumulation of protein aggregates and the body’s inflammatory response to the aggregates as well as the cell injury caused by the aggregates. Additional genes that contribute to aggregate formation are those of the autolysosomal system that control the accumulation of aggregates, which includes genes such as sequestosome 1 (SQSTM1), valosin-containing protein (VCP), lysosomal enzymes (glucosylceramidase beta 1,GBA1, cathepsins, etc.), lysosomal repair (LRRK2) and the exosomal pathway (ESCRT) [42,43].

## 3. Biological Condensates and Liquid–Liquid Phase Separation

A significant fraction of the genes linked to neurodegenerative diseases fall into the category of RBPs, which includes genes linked to ALS, FTD-TDP and SCAs [44]. MAPT also falls into the same category of proteins. RBPs constitute such a large fraction of the contributors to neurodegeneration as a result of the additive effects of several of their properties. The first major disease-related property is the reliance of RBPs on liquid–liquid phase separation (LLPS) to form biological condensates, which mediate the mechanism for their physiological actions; these condensates are also referred to as membraneless organelles [45,46,47,48,49,50,51]. RBPs control RNA localization and utilization by grouping together in loose complexes that contain RBPs and RNA. These proteins contain domains with a limited number of amino acid types, which are termed low-complexity domains (LCDs). These LCDs feature amino acids that interact with one another through hydrogen bonding that is supplemented by a moderately stronger interaction, termed Pi bonding. Importantly, the negatively charged RNA strands create an environment that promotes the formation of liquid droplets containing these RBPs. This process essentially coordinates RNA metabolism, allowing proteins and transcripts with related functions to consolidate, increasing their interactions and promoting functional interactions [48,52,53,54].

The reliance on phase separation presents a problem that becomes more apparent with aging and with chronic stress. Many of the disease-linked RBPs, such as TDP-43, ATXN2 and FUS, readily convert to insoluble aggregates. A fraction of RBPs change conformation in a manner that enables interactions characterized by β-pleated sheets [48,52,53,54]. RBPs differ in their threshold for converting from a dynamic phase-separated liquid droplet to stable oligomers and insoluble aggregates, some of which form β-pleated sheets. In healthy cells, misfolded proteins and oligomers are rapidly identified by chaperones, ubiquitinated and shunted for removal by the proteins that control catabolism of misfolded proteins, including SQSTM1 and VCPs [4,55,56]. However, aging or exposure to chronic stress impairs lysosomal function, which correspondingly reduces the ability to remove misfolded or oligomeric proteins and insoluble aggregates [57,58,59]. Thus, as we age or experience chronic disease, oligomers and insoluble protein aggregates accumulate. This propels a cycle that promotes neurodegeneration: (a) reduced proteostasis/catabolism increases the levels of oligomeric proteins and insoluble aggregates; (b) the oligomers and insoluble aggregates interfere with cellular functions, which causes stress, (c) general stress stimulates the translational stress response, which is associated with cytoplasmic translocation of nuclear RBPs (including TDP-43, Fused in Sarcoma RNA Binding Protein, FUS, T-Cell-Restricted Intracellular Antigen-1, TIA1 and Heterogeneous Nuclear Ribonucleoproteins A2/B1, hnRNPA2B1) [29,30,60,61]; (d) the cytoplasmic RBPs form SGs; and (e) persistent SGs bring together RBPs at high concentrations (micromolar), which increases misfolding, oligomerization and aggregation of RBPs [44]. The amount of oligomeric and insoluble aggregates that accumulate is influenced by the type of RBPs that are associated in SGs, genetic mutations or polymorphisms that reduce proteostasis, genetic mutations that reduce lysosomal function, as well as environmental stresses and aging. These factors act together to enhance the progression of neurodegenerative diseases.

The link between RBPs, SGs and disease mechanisms focuses attention on the functions of SGs. One of the main functions of SGs is to regulate protein synthesis and the translational stress response.

## 4. The Translational Stress Response (TSR)

The cell responds to stress by reducing protein synthesis to save energy. The large cellular protein content represented by ribosomal proteins (varying from 5 to 50%) might contribute to the tendency of a cell to reduce ribosomal protein production in response to stress [62,63]. Other proteins do not change as dramatically with stress, and the production of some proteins increases with stress, as will be described below [63].

The Eukaryotic Initiation Factor 2 (eIF2) pathway is the well-characterized mechanism for reducing protein synthesis. eIF2a normally binds to the RNA translation complex to initiate protein synthesis. Phosphorylation of eIF2A at S51 prevents this interaction, thereby terminating protein synthesis [64]. Four kinases control phosphorylation at S51: PERK, PKR GCN2 and HRI [64,65,66,67,68,69]. Each responds to a particular type of stress, ER, viruses, nutrient deprivation and oxidative stress. The phosphorylation allows eIF2a to inhibit eIF2B dimerization and block protein synthesis [68]. Dimerized eIF2B functions to compete with eIF5, allowing for the exchange of GTP for GDP [68,70,71,72]. Once primed by GTP, the multi-subunit EIF2 complex helps to initiate synthesis of proteins by ribosomes on a mRNA strand [68,70,71,72]. A decade ago, Peter Walter’s team discovered the compound ISRIB, which binds to eIF2b and stabilizes it as a dimer, making it permanently active, which strongly activates GTP exchange [70,71,72,73]. Activation of eIF2b circumvents eIF2a phosphorylation and inhibits the translational stress response (TSR), which disperses SGs [73]. Translational stimulation by ISRIB is protective in multiple models of neurodegenerative disease, including models of ALS based on TDP-43, AD based on β-amyloid or tau [74,75,76,77,78]. Treatment with ISRIB increases markers of synapses and synaptic plasticity. However, the treatment does not reduce markers of pathology (Aβ, TDP-43 or tau) and is not beneficial in all animal models of degeneration, which suggests that this pathway does not stimulate proteostasis or protein degradation [78,79,80]. Thus, the protection by ISRIB appears to occur by stimulating synaptic function. Denali, Calico/Abbvie and Bristol Myers Squibb developed analogs of ISRIB with improved brain penetrance. These compounds went into clinical trials, successfully passing through Phase I but subsequently failing in Phase II trials for ALS. Denali is pushing their compound into clinical trials for a rare disease, white matter degeneration, which is associated with mutations in EIF2B [81,82,83].

Whereas the eIF2 system acts as a central hub to modulate RNA metabolism, mTOR acts as a central hub to modulate protein synthesis. mTOR regulates ribosomal function and protein synthesis through phosphorylation of S6 Kinase [84]. mTOR also directly regulates protein catabolism by phosphorylating ULK1, Autophagy-Related Protein 13 (ATG13) and AMBRA1 to regulate autophagic flux [85,86]. Inhibiting mTOR reduces protein synthesis while at the same time increasing autophagic flux. If lysosomes are functioning well, then the increased autophagic flux helps to remove protein aggregates as well as recycles proteins for generation of more amino acid building blocks. Rapamycin and homologues fall into a general class of mTOR inhibitors, termed Rapalogs [87,88]. These have been tested in animal models of AD but so far have yet to show promise in clinical trials for AD [89,90]. Rapalogs have not garnered much traction in clinical trials because lysosomal function is thought to be impaired in elderly patients with AD [91]. Multiple studies demonstrate that autophagosomes accumulate in the AD brain [91]. This is thought to occur because reduced acidification of lysosomes prevents fusion of the autophagosome with the lysosome [91]. Absent such fusion, autophagasomes lack the actual enzymes that degrade their contents.

## 5. Parallel Translation Initiation Pathways Regulate the Chronic Stress Response

The Eukaryotic Initiation Factor 4 (eIF4) and Eukaryotic Initiation Factor 3 (eIF3) pathways also contribute to disease. Mutations in EIF4G1 have been shown to be associated with Parkinson’s disease in multiple studies, particularly in Asia; however, one study noted such EIF4G1 mutations in a cohort of neurologically normal Caucasians [92,93,94,95]. The mechanism underlying this association remains unexplored, but it is important to note that eIF2 interacts with the eIF4 complex as part of the translation initiation pathway [92]. The eIF3 complex operates in parallel with eIF2a to regulate the TSR, but has attracted relatively little attention. eIF2a phosphorylation occurs quickly in response to stress, and leads to reduced general protein synthesis. Ribosomal proteins are the largest group of proteins that are reduced in response to stress [65,96]. The decreased production of this class of proteins might reflect their abundance in the proteome (ribosomal proteins constitute ~5–50% of cellular proteins) and the large amount of energy expended on proteins synthesis. Stress is associated with reduced energy production, which demands a corresponding reduction in energy expenditure. Reducing protein synthesis is an obvious mechanism to reduce cellular energy expenditure.

The effects of eIF2a phosphorylation are transient. The eIF2 pathway begins to recover after one hour of stress with phosphorylation of eIF2a decreasing, protein eIF2b GTPase activity increasing and protein synthesis partially recovering [97]. Meanwhile, many parts of the integrated stress response remain active, despite loss of eIF2a phosphorylation. One pathway compensating for loss of peIF2a is phosphorylation of eIF3d, which occurs over a slower time course than phosphorylation of eIF2a, accumulating only after stress has occurred for at least one hour [98]. Phosphorylation of eIF3d enables protein synthesis of key ISR proteins, including synthesis of mTOR, PI3K and FOXO1 [98]. This particular “chronic stress” pathway is selective in that it responds to glucose deprivation but not serum deprivation, which raises the possibility that other translational pathways might be selective for disease-related stresses, such as the accumulation of protein aggregates [97]. The eIF3 complex identifies its RNA targets based on binding to mRNA sites in the 5’ promoter region that contains *N*^6^- methyl-adenosine (m^6^A) modifications [99]. Binding of eIF3 to the m^6^A modified RNA sites stimulates protein synthesis from noncanonical start sites [99]. Thus, eIF3 enables synthesis of some proteins during the TSR.

One final point concerns stress responses including the TSR. The regulation of the TSR varies by the type and duration of stress. Specificity of stress responses is exemplified by the selective phosphorylation of eIF3d in response to glucose deprivation but not serum deprivation and occurring in a delayed manner [98]. There are many other examples of selective responses to stress. For example, TDP-43 translocates into the cytoplasm upon proteasomal inhibition, while FUS translocates to the cytoplasm with DNA damage [100,101,102,103]. The composition of the resulting SGs varies in response to the particular stress and/or the particular cell type, although the down-stream consequences of these differences are poorly understood [104].

## 6. Stress Granules and Neurodegenerative Disease

**Figure 1:**
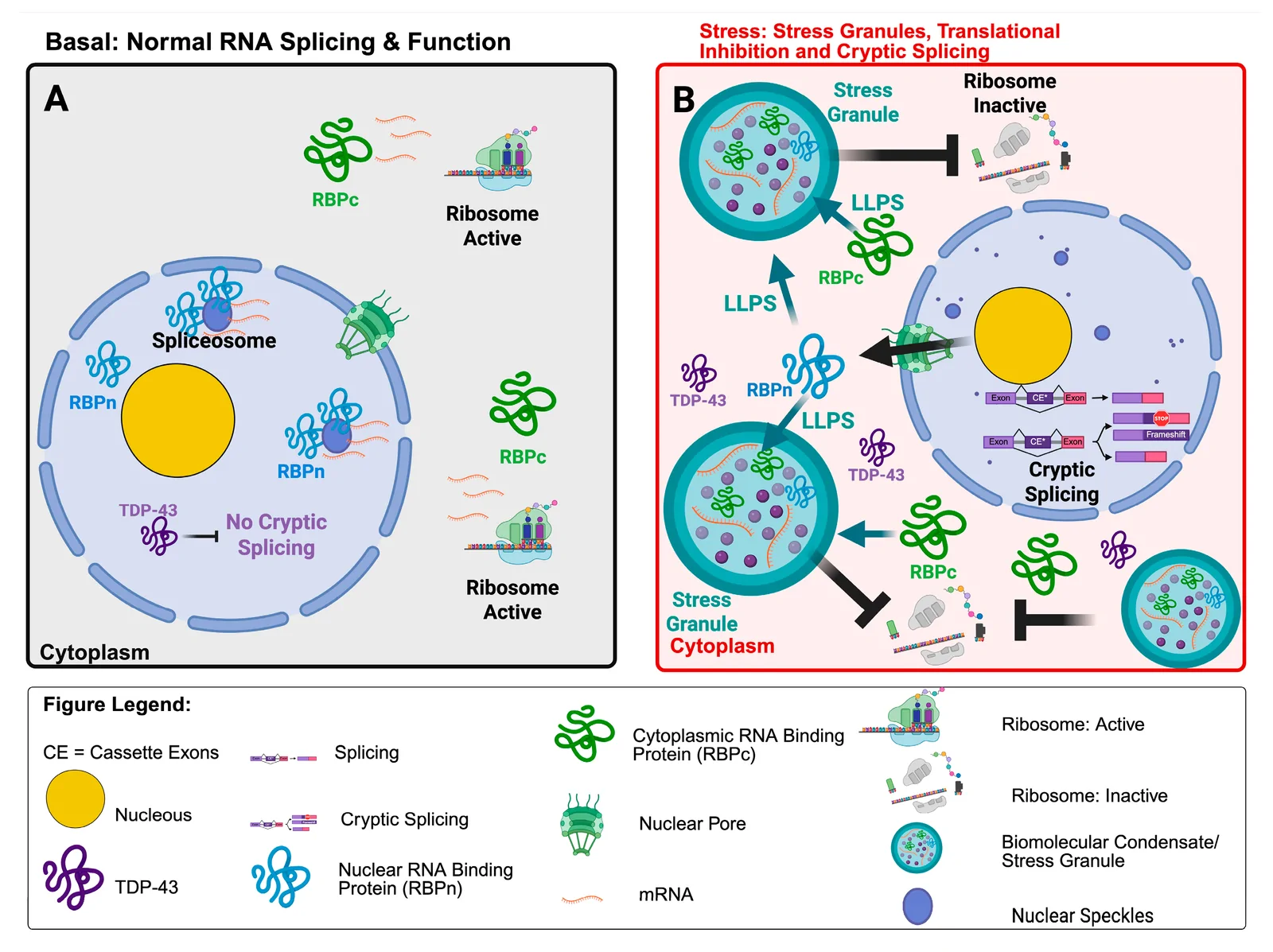
**Biology of the stress response.** (**A**) Under basal conditions, nuclear RBPs facilitate splicing in membranous organelles termed nuclear speckles and nuclear TDP-43 prevents deleterious cryptic splicing. Cytoplasmic RBPs facilitate protein synthesis in a process mediated by eIF2, 3 and 4. (**B**) Stress causes nuclear RBPs to translocate to the cytoplasm where they phase separate with cytoplasmic RBPs and mRNAs to form stress granules, which inhibit ribosomal protein synthesis. Reduced nuclear TDP-43 allows deleterious cryptic splicing which is thought to contribute to neurodegeneration.

RBPs are the signature proteins modulating the TSR (Figure 1). Stress induces post-translational modifications of RBPs, including arginine methylation and serine/threonine phosphorylation [47,105,106,107,108,109,110,111,112]. These modifications cause particular cytoplasmic RBPs, such as Ras GTPase-Activating Protein-Binding Protein 1 (G3BP1) and G3BP2 (selectively abundant in the brain), to begin to form membraneless organelles (MOs) through a process termed liquid–liquid phase separation (LLPS). Nuclear RBPs, such as TIA1, exit and also contribute to the nucleation (Figure 1). LLPS is mediated by LCDs of the RBPs that loosely associate through simple chemical interactions such as hydrogen bonding, Pi bonding and ionic bonding [28,30,113,114]. Although this process can be induced in vitro by adding crowding chemical (e.g., polyethylene glycol), the process in the cell incorporates multiple other elements. RBPs bind RNA through RNA recognition motifs [53,115,116]. RNA itself can phase separate, but the process occurs more readily with the RNA/RBP complexes because the RNA helps to increase the local concentration of RBPs and also provides a supportive ionic environment. Scaffolding proteins, such as CAPRIN1 and SERBP1, also contain LCDs and contribute to the process by acting as bridges for adjacent RBPs [117,118]. All of these elements work together, enabling LLPS in the complex, chaotic environment of the cell.

Phase separation of proteins and mRNA in response to stress enables the formation of SGs as regulatory structures (Figure 1). These SGs act to generally lower the amount of protein synthesis and resulting energy usage in response to acute stress and generally promote cellular resilience. This broad generalization masks a myriad of subtleties in the system. The eIF3 complex binds to translational promoters marked by m^6^A to stimulate the synthesis of proteins that help address the stress [99]. SGs can also serve as docking centers. For instance, SGs bind to lysosomes by docking with Annexin A11 [119]. They appear to use this docking for transport towards the synapse, perhaps delivering mRNA and proteins relevant to the stress response. Mitochondria are another organelle to which SGs dock, possibly for the same reason [120,121]. Finally, MOs, including SGs, appear to adhere to damaged regions of lysosomes, providing a temporary patch [122]. Recent studies show that the ESCRT machinery actively docks with damaged lysosomes to facilitate removal of the damaged area [123]. It seems possible that binding of SGs to damaged lysosomes might provide an alternative pathway for addressing lysosomal damage. These examples provide strong evidence that SG functions extend well beyond simple translational arrest and support cellular resilience.

The state of SGs, and indeed all MOs, varies with cellular conditions and the duration of phase separation [124]. Some of this is strongly connected to the physiological functions of MOs and SGs. MOs with high fluidity do not transmit signals across the MO [124]. For instance, a highly fluid MO cannot extend structural molecular changes occurring to a small set of molecules because the fluid will easily adapt to any change in the structure [124]. Increased vitrification MO (i.e., becoming less fluid) increases the transmission of structural changes. Changes in orientation or interactions of a molecule on one side of a vitrified MO will tug at the neighboring molecules, which will tug at their neighboring molecules, etc., causing the signal to “percolate” across the span of the MO [124]. The other extreme also applies. Solidification of an MO blocks signal percolation. Change in binding state for a molecule on one side of the solid will not transmit information across the structure because of the rigidity of the solid. Not surprisingly, many MOs in the cell appear to exist in a somewhat gelatinous, vitrified state, which readily transmits information. This vitrification is particularly relevant at the synapse, which exists as a large phase-separated organelle that exists to transmit information [125,126].

## 7. Disease-Linked Mutations in RBPs Increase Aggregation and Are Associated with Disease

MOs are hypothesized to contribute to neurodegenerative diseases, such as ALS and FTD-TDP43 because multiple RBPs that have been identified in MOs also have mutations that are linked to these diseases. The discovery of TDP-43 pathology in ALS and ALS-linked mutations in the genes TARDBP and FUS was followed by studies showing that TDP-43 and FUS incorporate into MOs in response to stress [127,128]. The disease-linked mutations tend to occur in LCDs that drive LLPS and the formation of MOs. My group discovered that TDP-43 pathology was associated with other SG proteins and that the disease-linked mutations increase the tendency of TDP-43 to form SGs [128]. Subsequent studies demonstrated that the mutations exhibit an even stronger effect on dissolution of TDP-43-containing SGs after stresses (such as arsenite) are removed [105,129,130,131]. SG biology, though, appears to represent only part of the pathophysiology of TDP-43. Studies have shown that TDP-43 en route to the formation of pathology appears to transit through SGs, rather than remaining in SGs and vitrifying [105]. Induction of TDP-43 pathology in cells over 24 hrs shows an initial localization to SGs followed by a subsequent shift toward a pathological inclusion separated from functional SGs [44,132].

Other RBPs with mutations linked to ALS or FTD also act in SG pathways, although it is important to note that SGs are a heterogeneous group of MOs that form in response to stress but whose function, composition and kinetics can vary depending on the type of stress. Mutations in the RBP TIA1 are associated with ALS and other proteinopathies in rare families [133,134]. TIA1 is a core SG-nucleating protein, analogous to G3BP1 and 2. It regulates both inflammation and neuronal signaling. The disease-linked mutations in TIA1 increase the tendency of the protein to aggregate; these mutations lead to SGs that have a reduced ability to disperse, which means they create SGs that are more likely to foster protein aggregation (Figure 1) [134,135]. There is an abundance of evidence linking TIA1 SGs to aggregation of Tau, but, surprisingly, the pathology of cases with TIA1 mutations shows TDP-43 pathology [134]. This unexpected result could occur if primary TIA1 aggregation elicits a signaling pathway that favors TDP-43 MOs/SGs. For instance, TIA1 is known to have a strong role in inflammation, and emerging studies link TDP-43 aggregation with strong inflammatory responses [136,137,138].

Other RBPs with ALS/FTD-linked mutations follow a pattern of pathology that is more consistent with a primary focus on SGs and subsequent aggregation. Mutations in FUS, TAF-15 and EWS that are linked to ALS or FTD all tend to increase the propensity of these proteins to aggregate, as well as increasing the accumulation of MOs and aggregates containing these proteins. One surprise is that these proteins can form amyloids that contain more than one type of protein. For instance, TDP-43 can form an amyloid that contains Annexin A11 [139]. The mechanism of toxicity for these aggregates remains unclear.

The pathophysiology for most of these aggregation-forming RBPs remains unclear. The exception is the pathophysiology of TDP-43, where current evidence suggests that the most important element of TDP-43 pathophysiology occurs on the path towards SGs but through a process that is independent of SG formation. Under basal conditions, TDP-43 mainly exists in the nucleus, where it functions to protect against cryptic splicing. Stress causes TDP-43 to translocate to the cytoplasm for reasons that remain unclear. This cytoplasmic translocation reduces levels of nuclear TDP-43, which allows cryptic splicing to occur. Many transcripts undergo cryptic splicing, but some of these produce transcripts that are deleterious to neurons and/or the associated reduction in the cognate transcript is detrimental. Two good examples of transcripts prone to cryptic splicing that is regulated by TDP-43 are the cytoskeletal protein STMN2 and the mitochondrial protein UNC13a, the latter of which is a known genetic modifier of ALS progression [34,36,140], Loss of either STMN2 or UNC13a is sufficient to cause neurodegeneration, and cryptic splicing of both transcripts is known to reduce levels of the cognate transcript while producing nonfunctional cryptic transcripts [141].

## 8. Tau and Stress Granule Biology

**Figure 2:**
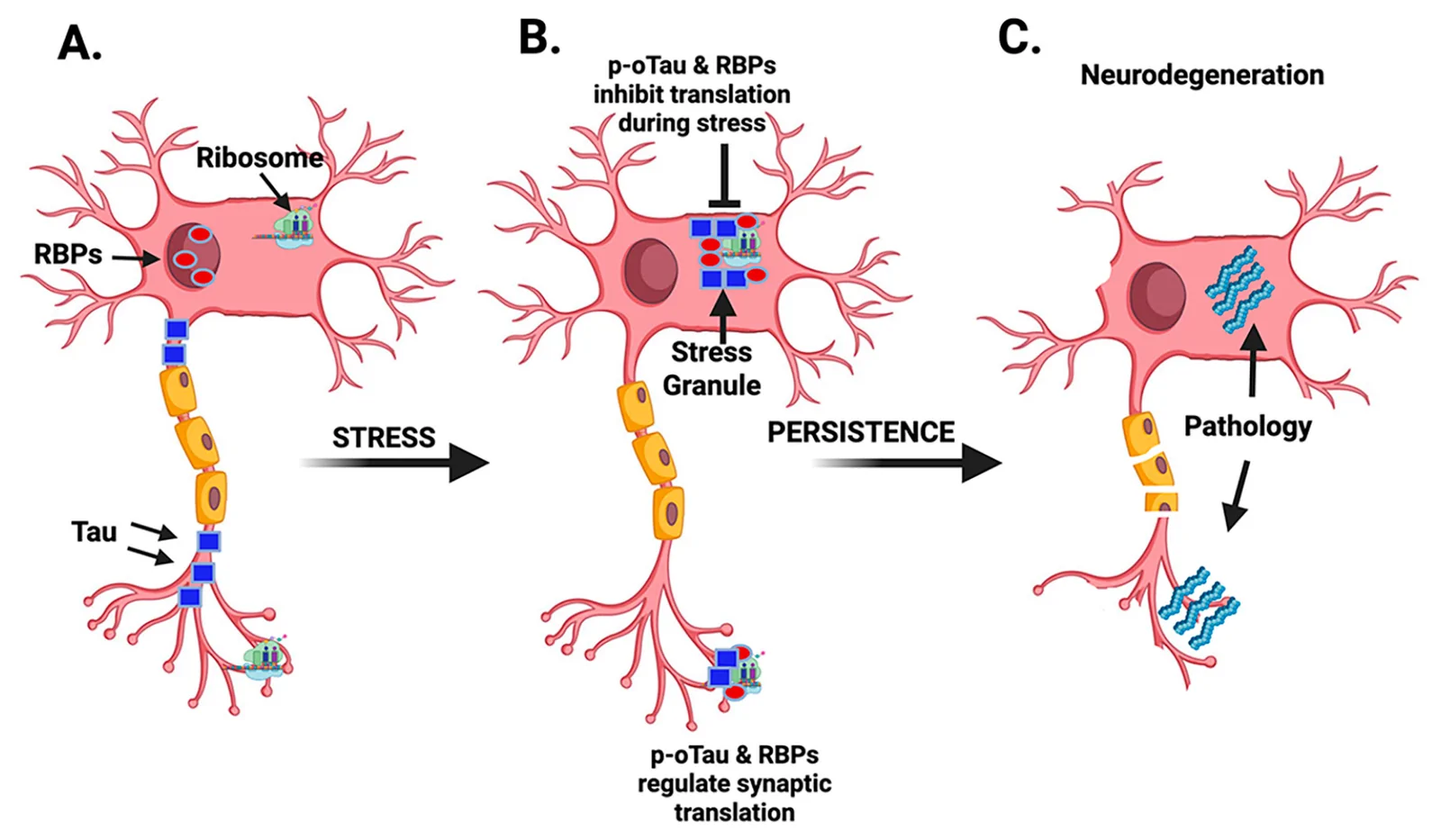
**The evolution of pathology in neurodegenerative diseases.** (**A**) Under basal conditions, many RNA-binding proteins (RBPs) exist in the nucleus, where they control processes such as transcription, RNA splicing, adenylation and RNA degradation. Tau exists mainly in the axon, and the ribosome functions at full capacity, producing proteins. (**B**) Stress causes many RBPs to exit the nucleus, where they can form biological condensates, which includes a category termed SGs. Stress also causes phosphorylation of Tau, which elicits two changes. Stress-mediated phosphorylation of Tau causes it to disengage from microtubules. Phosphorylation also causes rapid oligomerization of Tau. This phosphorylated, oligomeric tau (p-oTau) accumulates in the cytoplasm, binds to RBPs and helps to maintain and grow the SGs. The SG acts to inhibit protein synthesis by the ribosome. (**C**) Chronic diseases, such as neurodegenerative diseases, cause the SGs to persist. RBPs have a high propensity to aggregate, forming pathological protein aggregates from persistent SGs. Persistent SGs also promote the accumulation of p-oTau, which is toxic to neurons. With time, the p-oTau fibrillizes, contributing to the neurofibrillary tangles that characterize the intracellular pathology of AD.

Tau protein is abundant in neurons and also regulates SG formation (Figure 2). More tau gives larger SGs and less tau renders neurons resistant to SG formation in response to environmental stress, Aβ treatment and behavioral stress [60,61,142,143,144]. The phosphorylation state of tau also determines the size of SGs, with pseudo-phosphorylated tau yielding large SGs and pseudo-null phosphorylation yielding smaller (but more abundant) SGs [60]. In vitro studies of Tau demonstrate that it undergoes LLPS in the presence of RNA [145,146,147,148]. LLPS elegantly show the vitrification of MOs with time [28]. Incubation of Tau in solutions with the RBP TIA1 shows that Tau rapidly partitions into TIA1 LLPS droplets [28]. At first, Tau is miscible with the TIA1 and diffuses throughout the droplet, but over 60 min Tau begins to contract and exclude TIA1, forming a consolidated phase-separated species that is gel-like and vitrified [28]. This process does not occur in the absence of TIA1 and RNA; LLPS droplets containing only Tau remain highly dynamic [28,145,146,148]. The interactions between Tau on RBPs are specific to particular RBPs; Tau is miscible in LLPS droplets containing TIA1 and hnRNPA1, but not in droplets containing G3BP1, eIF4A1, eIF4E or DDX6 [28]. Importantly, the presence of TIA1 stimulates the formation of Tau oligomers and induction of the type of misfolded Tau observed in the brain from subjects with AD and related dementias [28,60]. These Tau oligomers are also more toxic to neurons than fibrillar tau (monomeric tau in its native conformation is nontoxic), similar to what is observed in the AD brain [28]. Thus, Tau interacts with SGs in cells and in vivo and with LLPS RBP droplets in vitro, and these biomolecular condensates (also termed MOs) serve as crucibles for the accumulation of toxic tau oligomers.

Why would the body do this? Tau is classically known as a microtubule-binding protein. Indeed, Tau forms a biomolecular condensate at the tips of microtubules, providing one potential explanation for the propensity of Tau to phase separate [146]. We hypothesize that Tau has added functions. Hyperphosphorylation of Tau (i.e., phosphorylation at serines that are in the region of prolines) by stress kinases is central to the pathophysiology of Tauopathies but also occurs in all infants as well as hibernating animals [149,150,151,152,153]. This phospho-Tau does not accumulate as pathological aggregates and likely serves a biological function. Phosphorylation stimulates rapid dimerization/oligomerization of Tau [154]. It should be noted that this is true both for human Tau and for mouse Tau, even though moue Tau has a much lower propensity to fibrillize than human Tau [155]. Many RBPs bind to oligomerized Tau, and we have explicitly shown that hnRNPA2B1 binds faster to phosphorylated oligomerized Tau than to native Tau [30,156,157,158,159]. These studies show that phosphorylated oligomeric Tau (p-oTau) enhances the formation of SGs in response to stress, while Tau removal reduces the formation of SGs containing TIA1 (but perhaps not TDP-43). These data lead to the hypothesis that Tau functions during stress to promote the TSR (Figure 2). In young animals with healthy proteostasis machinery (e.g., lysosomes, proteasomes), any fibrillar tau is rapidly removed; Tau in immature systems also appears to have different patterns of acetylation, which could be an additional factor that inhibits its fibrillization [160]. These combined data point to a strong role for Tau in regulating the TSR.

Evidence supporting a role for Tau in modulating translation is also accumulating in vivo. Tau interacts with ribosomal proteins, such as RPS6, and also binds small nuclear RNAs (SNOs) that are necessary for ribosomal synthesis [157,161,162]. Constitutive KO of Tau inhibits the stress response induced by chemicals or even by behavior (chronic unpredictable stress) [163]. Taken together, these data suggest a model in which stress-mediated phosphorylation of tau elicits rapid oligomerization. The p-oTau binds RBPs linked to the TSR, enhancing the formation of SGs. Phosphorylated Tau also acts at other levels to regulate translation, binding to RPS6 and binding to SNOs, thereby regulating ribosomal function [161,162].

## 9. The Role of SGs in the Pathophysiology of Tauopathies

Aging is associated with deterioration of proteasomal systems, lower efficiency of mitochondria, increased DNA damage and a more reactive inflammatory system. Each of these processes enhances the stress response. The result is that cells experience an increased abundance of protein aggregates, combined with more stress and impaired proteostasis. All of this leads to the accumulation of Tau pathology as oligomeric tau transitions to fibrillar tau (Figure 2). Studies of Tau in the human AD brain and in animal models of AD show pathological Tau co-localizing with RBPs in the early to mid-stages of tangle formation [30,61,158]. AD and FTD brains show colocalization of Tau with multiple different RBPs, including TIA1, hnRNPA2B1, Musashi, G3BP2, SRRM2, etc. [30,61,158,162,164,165]. The colocalization is greatest in stages II–IV, and is low in stages V and VI, where many of the neurofibrillary tangles have progressed to a mature form. The Tau present in these stress granules also co-localizes with RNA, consistent with the structures being active SGs. These results are consistent with a model in which phosphorylation of Tau leads to Tau oligomerization, and the p-oTau associates with RBPs and mRNA.

SGs have two general functions. The first is to sequester mRNA away from active ribosomes, thereby reducing synthesis of abundant housekeeping proteins, such as ribosomal proteins [44,66]. SG also serve as sites where particular stress-responsive proteins are synthesized. The mechanism for this synthesis is described above and relies on eIF3 identifying noncanonical start sites, in part identified by the presence of m^6^A modifications [97,98,99]. The relationship between pathological Tau and ribosomes is unclear, but what is clear is that phosphorylated Tau associates with ribosomal proteins, such as RPS6 [157,161]. Thus, the p-oTau in SGs appears to be subserving multiple functions.

Conversion of oTau to fibrillar tau results in reduced binding to many proteins, including RBPs. Proteins associated with end-stage pathology, such as Ubiquitin, SQSTM1 and VCP, show increased binding to fibrillar Tau [30,156,157,159,161]. This conversion effectively reduces the amount of free oTau in the neuron. Since oTau is the functional species, fibrillization of tau presents a mechanism to sequester it to make neurofibrillary tangles, which are structures that appear to be less toxic than oTau. Some evidence suggests that fibrillar tau could be toxic, for instance by “spearing” the nuclear and lysosomal membranes, but multiple papers using animal tauopathy models indicate that neurons with neurofibrillary tangles are actually more physiologically active and show fewer signs of degeneration than neurons without neurofibrillary tangles [41,166,167,168].

## 10. Conclusions

A fundamental question underlying our understanding of neurodegenerative diseases is the question of why protein aggregates accumulate in neurodegenerative diseases. In the absence of disease-linked mutations, they only accumulate as the effects of aging become apparent, such as reduced proteostasis and increased inflammation. Even with aging, most proteins do not form toxic fibrils; there are only a small number of proteins that normally form the pathological amyloids associated with disease, and these are proteins that exhibit biophysical characteristics that belie a propensity to aggregate. Mutations in any of these proteins that increase this tendency to aggregate also increase the risk of neurodegenerative disease.

The biology inherent to RNA metabolism and RBPs is one of the major pathways leading to disease-related protein aggregation (with lysosomal dysfunction being another important pathway). RBPs organize themselves through a process of LLPS forming MOs that regulate RNA metabolism. One type of MO that stands out is the SG, which forms in response to stress, an important element of every disease. SGs bring together RBPs and RNA at high concentration, which, when persistent, provides conditions that promote the formation of the amyloids that cause disease (Figure 1 and Figure 2). Increasing impairment of proteostasis that occurs with aging or disease can reduce the ability to remove misfolded, aggregated proteins from MOs. As the amyloids accumulate, they begin to self-template and propagate further amyloid formation, which then leads to disease.

This model provides a potentially powerful platform for the design of disease-related therapeutics. One compound, ISRIB and its analogs, was already advanced to clinical trials. Despite failure in ALS, other elements of the LLPS/MO/SG pathway are being pursued in industry, which could yield further therapeutic innovations for neurodegenerative diseases.

## Data Availability

No new data were created or analyzed in this study. Data sharing is not applicable to this article.
